# Chlorine Solutions for a Safe Method of Decontamination of Breast Pump Milk Collection Kits Before and After the Coronavirus Disease 2019 Pandemic

**DOI:** 10.3389/fnut.2021.574311

**Published:** 2021-03-04

**Authors:** Virginie Rigourd, Benali Mouadh, Joel Poupon, Jerome Langrand, Arnaud Goutard, Christine Droguet, Emmanuel Bille, Pierre Frange, Yasmina Bahri, David Pasquier, Alexandre Lapillonne, David Skurnik

**Affiliations:** ^1^Human Milk Bank, Hôpital Necker-Enfants Malades, Assistance Publique Hopitaux De Paris, Paris, France; ^2^Department of Neonatology, Charles Nicolle University Hospital, Tunis, Tunisia; ^3^Biological Toxicology Laboratory, Hôpital Lariboisière, Paris, France; ^4^Antipoison Center of Paris, Service de Pharmacie, Hôpital Fernand Widal, Paris, France; ^5^Police Headquarters Central Laboratory, Paris, France; ^6^Department of Microbiology, Hôpital Necker-Enfants Malades, Assistance Publique Hopitaux De Paris, Paris, France; ^7^INSERM U1151-Equipe 1, Institut Necker-Enfants Malades, Université de Paris, Paris, France; ^8^Institut Necker Enfants Malades, Université Paris Descartes, Paris, France; ^9^Hôpital Necker-Enfants Malades, Assistance Publique Hopitaux De Paris, Paris, France; ^10^EHU 7328 PACT, Imagine Institute, Institut Necker-Enfants Malades, Université de Paris, Paris, France; ^11^Watchfrog Laboratory, Genopole (France), Évry, France; ^12^Department of Neonatalogy, Hôpital Necker-Enfants Malades, Assistance Publique Hopitaux De Paris, Paris, France; ^13^Division of Infectious Diseases, Boston Children's Hospital and Harvard Medical School, Boston, MA, United States; ^14^INSERM U1151-Equipe 1, Institut Necker Enfants Malades, Université Paris Descartes, Paris, France

**Keywords:** COVID-19, breastfeeding, breast milk expression, milk banks, decontamination

## Abstract

To promote breast feeding and breast pumping is essential for the most vulnerable infants even if the current coronavirus disease 2019 (COVID-19) pandemic sanitary crisis imposes more stringent hygienic measures. As recommended by the Centers for Disease Control and Prevention, World Health Organization, and Milk Bank Association, “after each pumping session, all pump part that come into contact with breast milk should be appropriately disinfected.” The present study proposed different methods than can be used and focus on the safety analysis of chlorine solution (CS) in terms of residual hypochlorous acid (HCA) and total trihalomethanes (THM). We also performed an efficacy testing of the CS approach to decontaminate the devices used to collect the milk (breast pumps and bottles). The bacteriologic results of 1,982 breast pump milk samples collected in three different settings showed a major decrease of the microbial contamination using either sterile device or decontamination with CS compared to a simple soap washing. The main messages from our study are to propose a guideline for the safe use of CS and to define situations when breast pump decontamination might be necessary: vulnerable babies for which sterile device is recommended; special circumstances, for example the current COVID-19 pandemic; special situations, for example women living in precarious conditions; or women pumping their milk at work but that would have low or no access to boiled water. Overall, cold decontamination reduced losses of milk for bacteriological reasons in human milk banks and may also be interesting to prevent horizontal contamination by virus like COVID-19.

## Introduction

A crisis situation such as the coronavirus disease 2019 (COVID-19) pandemic has changed the breastfeeding patterns of vulnerable neonatal hospitals (1). Despite the recommendations aiming at fostering the mother–child bond even in case of mothers infected by SARS-CoV-2, many mothers have to do breast pump feeding (2). One barrier is the prohibition or limitation of visits by parents to neonatal units to reduce the transmission of COVID-19 to caregivers and inter-family. This barrier measure in case of confirmed infections with COVID-19 can last for 14 days and at least 7 days after the disappearance of the symptomatology (3). In addition, several induced prematurities were motivated by respiratory distress in COVID-19 pregnant women then unable to breastfeed their newborns. These different new barrier measures and clinical situations imposed by organizations strengthen mothers' milk collection and transport to secure the enteral nutrient of their own child.

Breast milk is not sterile and human milk microbiota has multiple benefits for the health of the breastfed child (4–6). However, it can sometimes contain pathogenic germs, and the collection and storage of milk could increase the prevalence/concentration of environmental germs like bacteria and virus (7–10). Cases of bacterial infection following the use of raw milk have been published (9). Rejection for bacteriologic contamination is the most important risk for human milk banks, which is why they have special guidelines on hygienic measures and a cold chain for the collection, the treatment, and the storage of human milk (11, 12). The balance between bacterial safety and the risk of milk shortage is narrow as mentioned by Lewin et al. (13) and could be compromised in a crisis situation such as the COVID-19 pandemic (14). In previous emerging coronavirus outbreaks—SARS-CoV (2002–2003) and MERS-CoV (2012–2015)—no cases of vertical transmission were reported, and the virus has been detected in breast milk in only one case, whereas there is evidence of a significant secretory-IgA-dominant SARS-CoV-2 immune response in human milk following recovery from COVID-19 (data in press). While recent studies during the COVID-19 pandemic described early neonatal contamination, they also suggested a predominant role of the post-natal airborne route for these infections (2, 15, 16). It is still not clear whether the virus can or cannot be transmitted through breast milk. Even when RNA is detected in breast milk, it is not proof of viable and infective virus. Therefore, the use of maternal milk, directly by breastfeeding or after milk collection (use of a pump), has been recognized as possible during the COVID-19 pandemic and is recommended by the majority of national (CNSF, CNGO, and SFP) and international (WHO and EMBA) institutions (17, 18).

However, during this pandemic, for all hospitalized newborns whether their mothers are infected by SARS-CoV-2 or not, strict measures to prevent airborne and manual contamination during the prodigy care to her baby, breastfeeding, and milk collection have to be respected. Most human milk bank guidelines emphasize the importance of appropriate breast pump kit cleaning but do not specify the method of choice to reduce the bacterial and COVID-19 contamination. As a first step, regular cleaning and disinfection of affected surfaces with an ordinary household disinfectant containing a 1% diluted chlorinate solution appears as an essential barrier measure. However, in case of use of a pump, a second and equally important step has to follow: bio-cleaning procedures for the pump and its accessories with a virucidal detergent/disinfectant. Responding to these criteria and easy to use are chlorine pellets that can be diluted to form a chlorinated solution (CS) (19). In England, CS are considered as medical devices intended for surface disinfection in contact with foodstuffs with their regulations still being evaluated. However, in France, CS are not subject to the same regulation, and once their safety as barrier measure is evaluated, the use of CS could be scaled up, without going through the full and lengthy process applied to medical devices. This would provide a simple solution to numerous mothers to improve the safety of their maternal milk.

Once diluted, the sodium troclosene component releases hypochlorous acid (HCA), which is a highly reactive substance with biocidal properties. Upon contact with milk, HCA can react and form trihalomethanes (THM). These THM (such as chloroform, bromoform, dibromochloromethane, and bromodichloromethane) are a group of widespread and mildly lipophilic compounds with potential carcinogenic or reproductive toxicity (17). No study has investigated the amount of hypochlorous acid (HCA) or trihalomethane (THM) residual concentrations after use of CS as a method of decontamination of breast pump milk. The aim of this study was thus to evaluate the safety of decontamination of breast pump milk collection kits with CS helping to fill in an important gap in the care of some of the most vulnerable newborns in the hospital.

## Materials and Methods

We conducted a safety study of CS used for decontamination of breast pump milk collection kits in collaboration with the Paris Poison Center, the Biological Toxicology Laboratory of Lariboisière Hospital (Paris), and the Paris Police Headquarters Central Laboratory (LCPP).

Milton® sterilization tablets used in our human bank milk (Rivadis Laboratory, Louzy, France) contain 780 mg of sodium dichloroisocyanurate or troclosene sodium (C_3_Cl_2_N_3_NaO_3_) per tablet (19.5%). Sodium associated with troclosene represents ~11% in mass of the total sodium in the tablet.

### Samples of Woman's Milk

Breast milk was collected at a regional milk bank. All breast milk samples collected were stored in a refrigerator (+4°C) for a maximum of 48 h before being frozen at −18°C. When milk could not be delivered because it was considered non-conformer, instead of being discarded, we proposed to the mothers to use it for research. The control bottles were sterile water. The study was approved by the local ethics committee (i.e., CPP Ile de France II), and informed consent was obtained from all women participating to the study.

### Decontamination Procedure

The bottles were pre-cleaned with liquid soap, rinsed with clear water, and then decontaminated with CS. The CS decontamination of equipment is carried out after dilution of troclosene sodium tablets to constitute a CS usable for 24 h. The recommended dilution is one tablet for 5 L of water. The decontamination is effective after cleaning and a contact of 15 min in the CS, and the decontaminated material should be well-drained before use. The solution shall be kept at room temperature.

### Evaluation of Residual HCA Concentrations

The biocidal agent released by the hydrochlorinated solution is hypochlorous acid, but this substance is extremely reactive and therefore impossible to dose. The best way to deduce residual concentrations is to measure sodium concentration. Thus, sodium is a good reflection of the residual concentration of HCA (Bunsen's method). Eleven percent of the measured sodium comes from isocyanurate. As only a part of total sodium came from troclosene sodium, and after mass conversion, 1 μg of total sodium measured corresponded ~0.05 μg of HCA.

The concentration of sodium was measured by inductively coupled plasma mass spectrometry (ICP-MS) at the Biological Toxicology Laboratory of Lariboisière Hospital.

We prepared not only several solution trays with both the recommended dilution (one tablet for 5 L of water) but also three other dilutions (two, three, or four tablets for 5 L of water) to mimic dilution errors. The bottles used to collect the milk were soaked for 15 min in the different solutions. After draining, the bottles were filled with increasing volumes of milk (20, 50, 100, or 200 ml).

### Measurement of Total Trihalomethanes

The internal calibration assay of the THM was performed by gas chromatography coupled to mass spectrometry on the equilibrium vapor with the samples, supplemented with sodium thiosulphate, previously steamed according to the MOP0634 standard, at the Paris Police Headquarters Central Laboratory. The internal standard used is toluene-d8, which corrects the differences of responses of the analyses related to possible disturbances of the analytical system (Trihalomethanes in Drinking-water https://www.who.int/water_sanitation_health/dwq/chemicals/THM200605.pdf). Sodium thiosulfate stabilizes the sample by avoiding the formation of chloroform from free chlorine before and during the analysis process. The dosage was made from bottles decontaminated with a CS, diluted as recommended, well-drained, and filled with increasing volumes of mother's milk (20, 50, 100, or 200 ml). Two bottles of mother's milk (20 and 200 ml) without prior decontamination and two 100-ml sterile water bottles with and without decontamination were analyzed as controls. Control samples consisted to analyze (i) two bottles filled with mother's milk (20 and 200 ml) without prior decontamination, (ii) one drained CS-decontaminated bottle filled with 100 ml of sterile water, and (iii) one bottle filled with 100 ml of sterile water without prior decontamination.

### Dripping

The effect of dripping and draining efficacy was compared between 10 different operators using the standard dilution of Milton® and bottles filled with 100 ml of milk. Sodium was measured in each milk sample. Operators were randomly selected from the staff working in the milk bank and the baby bottle store.

### Mother's Milk

All milk samples used were from milk donated to the regional lactarium of Ile de France, which would have been rejected as non-complying with the milk bank guidelines (https://association-des-lactariums-de-france.fr).

An authorization from the Committee for the Protection of Persons Ile de France II has been obtained (N°APHP180022, IDRCB: 2018-A02069-46) and informed donors have agreed that their milk will be used for research purposes.

### Bacterial Analysis of Milk Before Pasteurization

#### Sample Preparation

The seeding of a given volume of milk on the usual medium allowed the number of bacteria present per milliliter to be counted in pre-pasteurization milks. This method followed the recommendations of the decree (Decision of December 3, 2007, defining the good practice rules according to paragraph 3 of article L. 2323-1 of the French Public Health Code NOR: *SJSM0722015S*). For the analysis of the milk before pasteurization, a sample of 3 ml per batch was taken once the bottles of the same batch emptied and filtered through the colander in the 1- to 4-L canister. The milk was homogenized with a sterile pipette; then, the volume of milk was taken with a pipette using a PepSy automatic sample. Pre-pasteurization samples were anonymized, identified by a label, and placed in a collection bag, which was placed in an insulated pouch kept at 4°C before being sent to the laboratory.

Upon arrival at the laboratory, the milk was seeded as quickly as possible and stored. The milk tubes were placed in doorways, and in a second door, tubes containing 5 ml of saline water were also placed. The doors were then placed in the KIESTRA seeding automaton. The two types of crop media (SAIDE medium and blood medium) were also deposited on the automaton, which put a label identical to that recorded by the laboratory to ensure identification and traceability. The program registered specifically for the lactarium was set in motion.

#### Sowing Pure Milk

All milk was grown on blood media and SAIDE, which were incubated in aerobiosis at 35°C for 48 h. This seeding was done using the automaton.

The automaton then placed a drop of milk of 10 μl, previously homogenized by its care, on each medium with the help of a cone, and then a small, sterile, disposable metal ball was used to carry out the seeding as below:


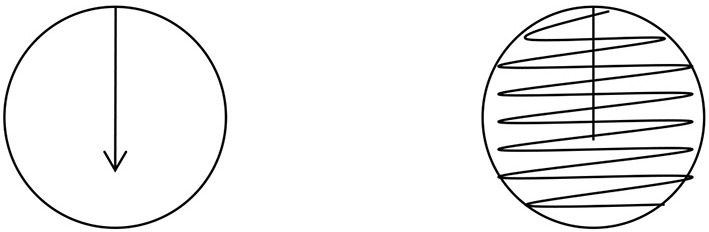


#### Sowing Diluted Milk

At the same time, the automaton was diluted to 1/1,000th (5 L of milk in 5 ml of water) in the saline water tubes placed for this purpose. The automaton made the same seeding from the inoculated water tube as for pure milk on blood agar (10 μl per box).

#### Interpretation of Cultures

The total flora was counted on pure and diluted blood media. A colony corresponded to 10^2^ UFC/ml in the starting milk for the pure seeded blood medium and 10^4^ UFC/ml for the diluted seeded medium. *Staphylococcus aureus* was researched specifically on SAIDE medium. In case of positivity to *S. aureus*, a quantification was made: a colony corresponded to 10^2^ UFC/ml in the starting milk. The identification of *S. aureus* was confirmed by MALDI-TOF mass spectrometry. No antibiotics or strain preservation were performed. Culture with more than 10^6^ bacteria or more than 10^4^ *S. aureus* was considered as contaminated and could not be used.

## Results

### Evaluation of Residual HCA Concentrations

**Figure 1:**
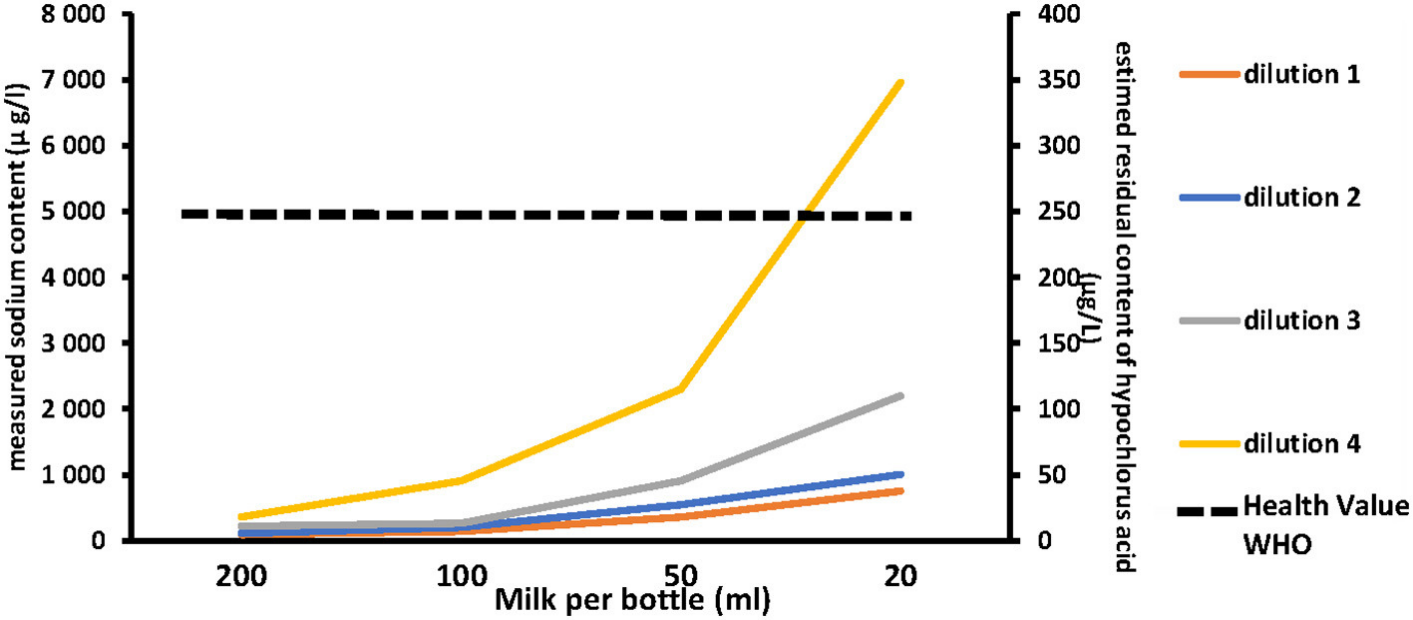
Left axis: measured sodium. Right axis: residual content of hypochlorous acid in milk according to the chlorine solution dilution method and the milk volume per bottle (from the right to the left side of the abcis the smaller volume to the higher on 200 ml of milk). Dilution 1: one tablet of Milton® for 5 l of water (Recommended dilution). Dilution 2: two tablets of Milton® for 5 l of water. Dilution 3: three tablets of Milton® for 5 l of water. Dilution 4: four tablets of Milton® for 5 l of water. 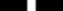 Dashed line represents the health value recommended by World Health Organization (5,000 μg/L).

**Figure 2:**
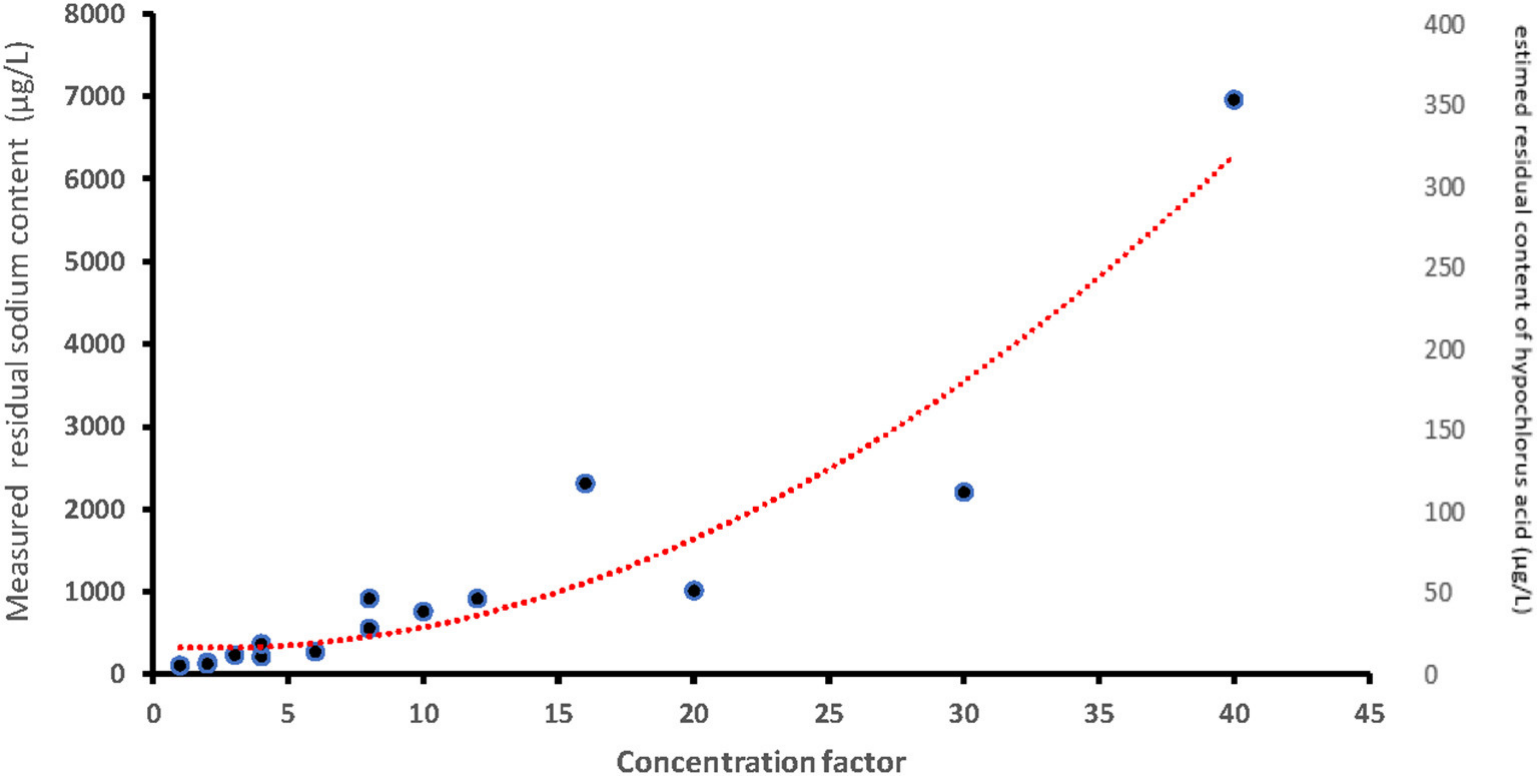
Left axis: values of the residual content of sodium measured depending on the concentration factor. Right axis: estimated residual content of hypochlorous acid (based on the residual sodium content measured). Concentration factor: factors which take into account the number of Milton® tablets for 5 l of water and volume of milk per bottle. Concentration factor is one when one tablet of Milton® was diluted in 5 l of water (recommended dilution) and the bottle was filled with 200 mL of milk.

Figure 1 illustrates the influence of the CS dilution method and milk volume introduced per bottle on the estimated HCA concentration in milk. The residual estimated concentration of HCA increased either by increasing the concentration of the chlorine solution or by decreasing the volume of milk contained in the bottle. Figure 2 shows the correlation between the dilution of CS, the milk volume in baby bottles, and the residual concentrations of HCA. Concentration factor takes into account the number of Milton^®^ tablets for 5 L of water and the volume of milk per bottle. Concentration factor is 1 when one tablet of Milton® was diluted in 5 L of water (recommended dilution) and the bottle was filled with 200 ml of milk. Under the usual dilution conditions (one tablet for 5 L of water), the residual estimated concentration of HCA in the milk was below the WHO guideline value for free chlorine in drinking water organoleptic and irritation threshold defined by the WHO (5 mg/l) (https://www.who.int/water_sanitation_health/dwq/chlorine.pdf) whether the bottles are filled with 200, 100, or even 50 ml. This threshold was reached only in the worst-case scenario consisting in the use of four tablets in 5 L of water for decontamination followed by an addition of 50 ml of milk in the previously drained bottle (smallest dilution).

### Measurement of THM

THMs are chloroform, bromodichloromethane, dibromochloromethane, and bromoform. These compounds can arise from disinfection products when chlorine interacts with organic matters such as the ones present in the maternal milk (20). The maximum threshold accepted for total THM in drinking water in France has been set at 100 μg/l (TDI of 150 μg/kg of body weight) (decree of 11th January 2007 relating to the limits and references of quality of raw water and water intended for human consumption modified by the decree of 9th December 2015 and the decree of 4th August 2017).

Table 1 represents THM concentrations in different samples of breastmilk and sterile water, with and without CS. THM levels remained below the toxic threshold regardless of milk volume. Of the four THMs, only the concentration of dibromochloromethane was influenced by the volume of milk.

**Table 1:** Trihalomethanes concentrations in different samples of breastmilk and sterile water (“control” bottle), with (yes), and without (no) Milton used with standard dilution (one tablet for 5 l of water).

	**Bottle 1**	**Bottle 2**	**Bottle 3**	**Bottle 4**	**Bottle 5**	**Bottle 6**	**Bottle 7**	**Bottle 8**	**Bottle 9**	**Bottle 10**
Volume (mL)	100	20	200	100	20	20	50	50	100	200
Solvent	Sterile water	Breast milk	Breast milk	Sterile water	Breast milk	Breast milk	Breast milk	Breast milk	Breast milk	Breast milk
Milton	No	No	No	Yes	Yes	Yes	Yes	Yes	Yes	Yes
Chloroform (μg/L)	<0.2	<0.2	<0.2	<0.2	<0.2	<0.2	<0.2	<0.2	<0.2	<0.2
Bromoform (μg/L)	<1	<1	<1	<1	<1	<1	<1	<1	<1	<1
Dibromochloromethane (μg/L)	<0.2	0.23	<0.2	<0.2	0.31	0.36	0.24	0.22	<0.2	<0.2
Bromodichloromethane (μg/L)	<0.2	<0.2	<0.2	<0.2	<0.2	<0.2	<0.2	<0.2	<0.2	<0.2
Total trihalomethanes (μg/L)	<1.6	<1.63	<1.6	<1.6	<1.71	<1.76	<1.64	<1.62	<1.6	<1.6

The concentrations of chloroform, bromoform, dibromochloromethane, and bromodichloromethane remained low in the different milk bottles after decontamination with a properly diluted CS. Total THM levels were below the toxic threshold regardless of milk volume in decontaminated bottle (20).

### Effect of Dripping Efficacy

**Figure 3:**
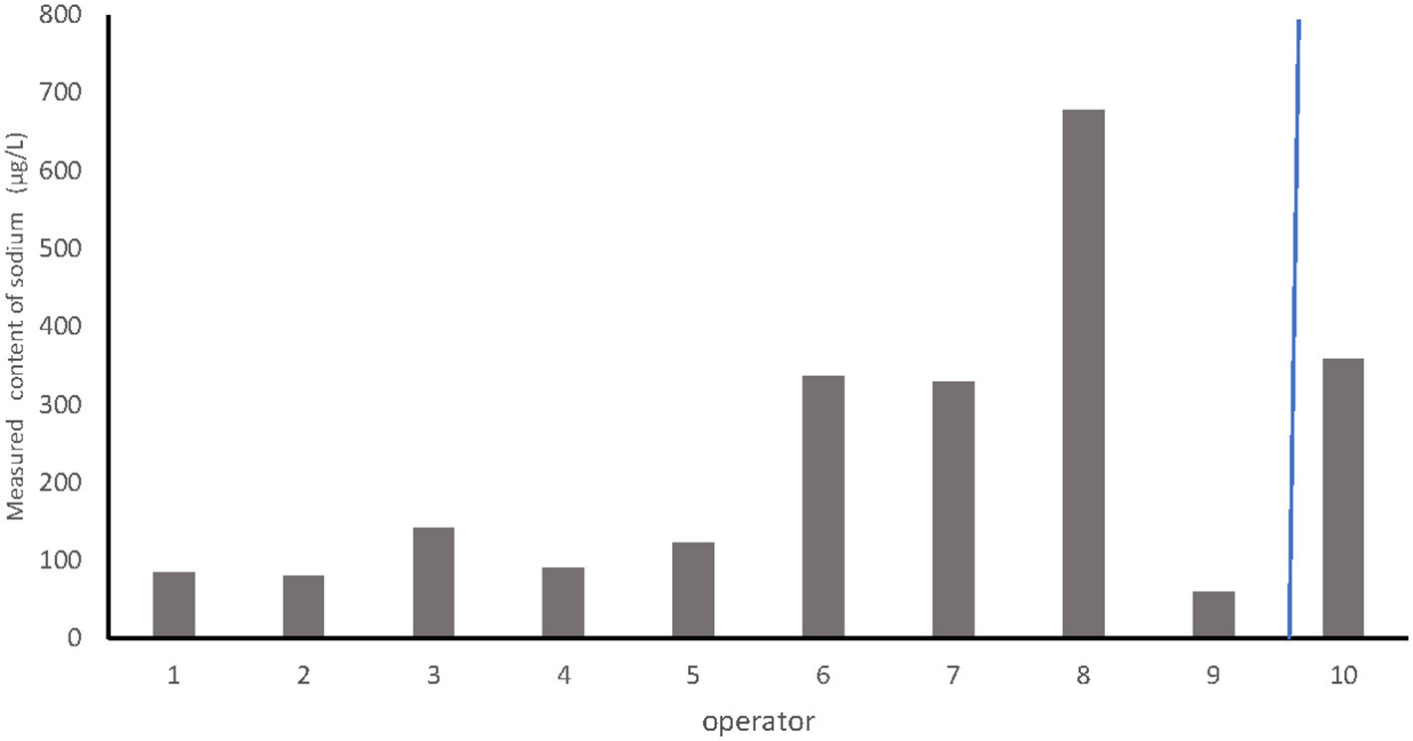
Comparison of the effect of dripping on the estimated content of hypochlorous acid between different operators.

Despite the use of the same dilution of CS obtained under the recommended protocol and the same volume of milk per bottle, the residual content of HCA ranged from 60 to 678 μg/l depending on the operator (Figure 3). The median was 312 μg/l. The organoleptic threshold was exceeded by only one operator.

### Efficacy Study

**Figure 4:**
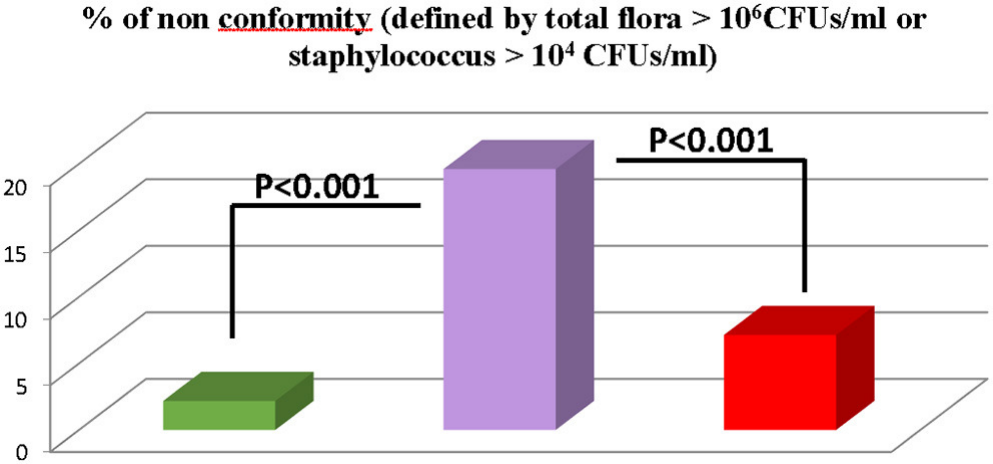
Comparison of bacteriologic results of 1982 breast pump milk samples collected in three different settings. 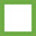 group 1 (*n* = 1,466) donors at home with device decontaminated with boiled water of chlorine solution. 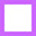 group 2 (*n* = 244) donors in other hospitals using neither decontaminated nor sterile devices, just soap cleaning of the devices and without washing the motor breasts. 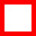 group 3 (*n* = 182) donors at the Necker hospital using sterile devices.

As shown Figure 3, we compared the bacteriologic results of 1,982 breast pump milk samples collected in three different settings: (1) at home with device decontaminated with boiled water or chlorine solution (group 1, *n* = 1,466); (2) at the Necker Hospital using sterile devices (group 2, *n* = 182); and (3) in other hospitals in the Paris area as they were using neither decontamination nor sterile devices, just soap cleaning of the devices and without washing the mother breasts (group 3, *n* = 244). Non-conformed milk (defined as total flora >10^6^ CFUs/ml or *Staphylococcus aureus* >10^4^ CFUs/ml) were significantly more found in the third group than in groups 1 and 2: 20% vs. 2 and 7%, respectively (Figure 4, *P* <0.0001). No difference was found in group1 between mother using boiled water vs. mother using CS (2% contamination in both subgroups).

## Discussion

Very premature infants cannot be breastfed directly by their mothers and will receive for many weeks their mother's milk in enteral nutrition, and when their own mothers' milk is not disponible, human milk collected, qualified, and pasteurized must be used (21, 22). Because of the fragility and the high susceptibility to infection of these newborn (prematurity or pathology), one of the questions in interest for health professionals is to reduce the risk of bacterial and viral contamination. Thus, milk collection and storage conditions need to be specifically controlled for (i) neonatal intensive care unit (NICU) to prevent raw milk contamination and infection diseases and (ii) milk bank to limit bacteriological rejection and prevent milk shortage (17, 23).

Previous studies have reported that when simple hygiene rules were followed for breast milk expressed, there was a decreased risk of contamination (24, 25). Recently, it has been shown that appropriate decontamination of breast pump milk collection kits was critical to obtain safe milk for infant and to avoid discarding donor human milk (20). Actually, most of preterm babies are fed by their own mothers' milk (26), which is not pasteurized, therefore increasing the risk of microbial contamination. This risk is also increasing in settings like the NICU because different members of the team will manipulate both the bottles and the milk. An additional risk of contamination is driven by the storage of the bottles at room temperature allowing bacteria and maybe viruses such as SARS-CoV-2 to grow. For all these reasons, both the environment (including the surface used to prepare and collect the milk) and the device must be cleaned. Our study performed in milk bank and testing the efficacy of the CS approach to decontaminate the devices used to collect the milk (breast pumps and bottles) is demonstrative. The bacteriologic results of 1,982 breast pump milk samples collected in three different settings are significantly different. We found the levels of bacteriological contamination, from the lowest to the highest levels, in the following: (1) at home mothers using either device decontaminated with boiled water or chlorine solution, with no difference between mothers using CS compared to mothers using boiled water; (2) at the Necker Hospital using sterile devices but with a lot of handling by different persons; and (3) other hospitals from the Paris area that were using just soap to clean off their devices, without washing the mother's breasts. Significantly more contaminated milk was found in this last group.

It was demonstrated that steam decontamination using a microwavable bag after washing resulted in a lower proportion of discarded DHM (1.3 vs. 18% *p* <0.001) (20). French Food Safety Agency therefore recommended sterile single-use or autoclavable sterilized or bacteriological clean breast pump milk collection kits for mothers in hospital. The discrepancy between hospital settings where human milk expression would be done in accordance with these strict conditions and the community where human milk expressed at home would be done using non-decontaminated breast shield is utterly divergent. The major COVID-19 pandemic that is still very active as of the writing of this paper has worldwide reinforced the mandatory aspect of some barrier measures, pushing significantly the necessity to have a safe and efficient decontamination protocol for both hospital and community settings.

Refusing milk expressed at home while privileging only milk drawn in hospital to prevent environmental contamination would not be an ideal choice as non-hospitalized mothers cannot ensure the daily ration of human milk during the newborn stay at the hospital.

Overall, there is a real need to help non-hospitalized mothers in their means to decontaminate their milk collection equipment. There are two major methods of decontamination: a hot one consisting in boiling water in a pan for 20 min, pressure cooker for 10 min, steam, or microwave sterilizers, which constitutes by far the most widespread method of decontamination; and a cold one, not only less common but also less time-consuming and probably easier to apply, using chlorine solution (CS) (27–29).

**Figure 5:**
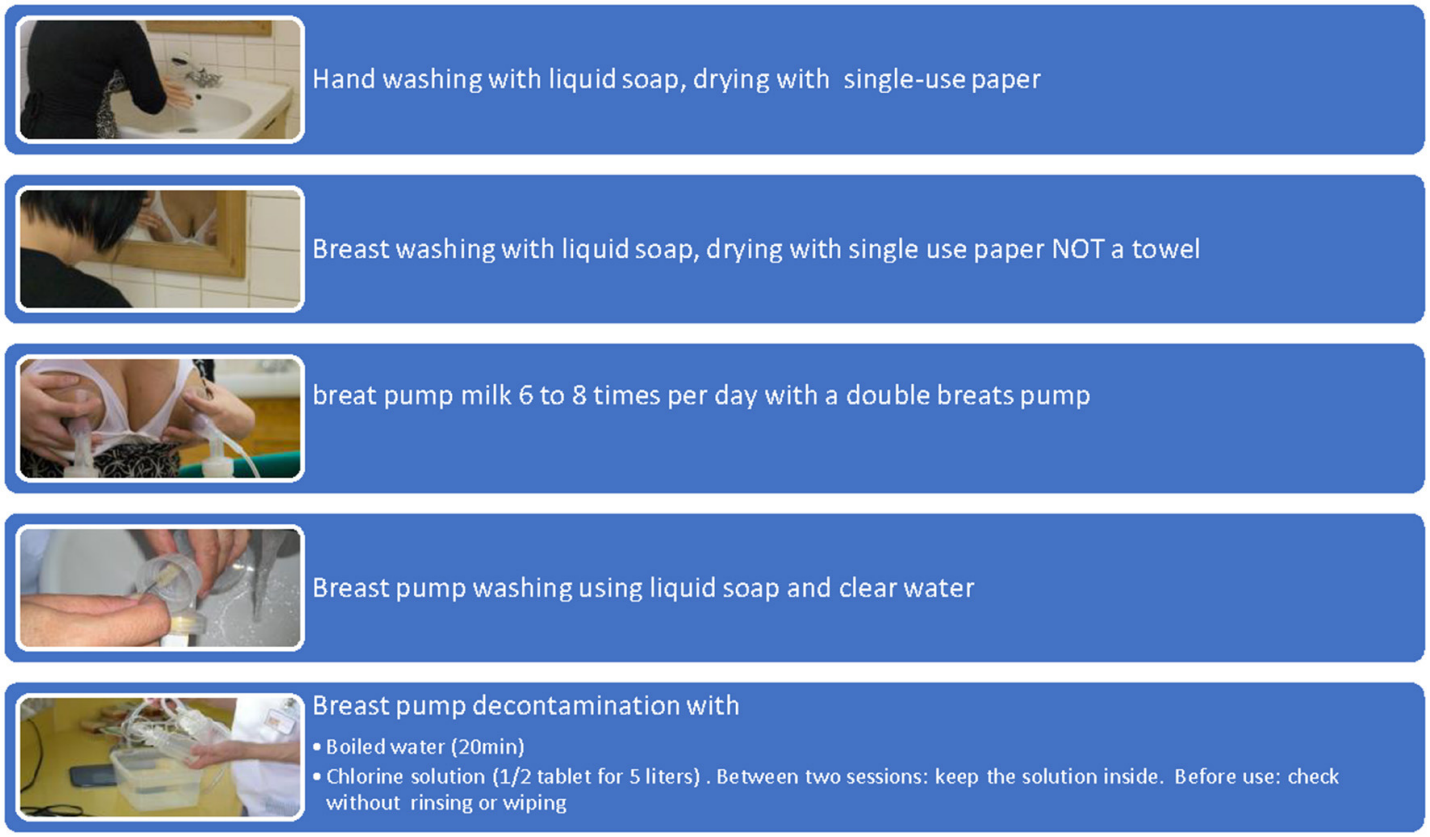
Step by step scheme for breast pump decontamination.

Figure 5 is an illustrated scheme of the decontamination (step by step) of a breast pump kit with CS.

Within these different means of decontamination of the milk collection equipment, microwavable bag does not seem to be appropriate against contamination by the SARS-Cov-2 virus because of the heterogeneity of the method and the lower temperatures applied. However, Chin et al. (30) confirm the destruction of SARS-Cov-2 at 56°C for 30 min. Boiling water might still be an efficient method of decontamination as already shown for SARS (31).

The approach presented here and based on chlorine solution has an excellent antimicrobial activity against bacteria, fungi, and viruses (32). Proofs of efficacy to European norms are bactericidal activity in 5 min (EN1040) including Methicillin-resistant *Staphylococcus aureus*. CS is also effective on *Listeria spp., Salmonella spp*., and *Campylobacter spp*. It is fungicidal in 15 min (EN1650, EN 14562) on *Candida albicans*. It is virucidal in 15 min on poliovirus type 1 and adenovirus type 5 according to the protocol EN14476 + A1. It is also effective on rotavirus, influenza A virus subtype H1N1, and coronavirus. CS at pH 7.2 has an effect on vegetative forms and spores of *Bacillus cereus* (30–32). Two cases of raw milk contamination were published while the breast shields were decontaminated with CS (33, 34). However, these results should be interpreted with caution as several factors must be considered: prior cleaning (essential to eliminate milk deposits), compliance with dilution, wiping after, or rinsing with contaminated water have been related. There are no randomized double-blind studies on the subject and no data on the number of infections avoided using similar equipment, as described in the procedure of this paper, or not.

Lucas (35) have proven the efficacy of CS in reducing milk contamination before pasteurization but mentioned the possibility of sodium contamination by chlorine solutions, but this link is theoretical, and no case has been reported (27). Concerning the risk of intestinal mucosa irritation and neurodevelopmental disorder mentioned by some authors, no causal link has been demonstrated under the usual conditions of use of CS (35, 36).

Our study has shown that, under the recommended dilution conditions, the estimated residual concentration of HCA is well below the WHO guideline value for free chlorine in drinking-water so that no sanitary effect is expected. Only in worst-case scenario with use of four tablets, and when the decontaminated bottle is filled with smaller volume of milk, the AHC levels exceed this threshold. The conditions under which milk is extracted do not expose small volumes of milk to CS. Atkinson et al. reported the average donation by hospitalized mothers providing a general overview of volumes collected (36). Observed cases show that on day 4, a mother who has given birth prematurely, and undergoing suitable initiation and stimulation of her lactation, supposedly supplies at least 200 ml milk a day collected in six samples minimum per day, namely more than 15 ml of milk per breast in each session of milk collection. From the 7th day, the volume produced is expected to increase to ~400 ml, constituted by a minimum of 30 ml per breast at each collection (37). However, at the colostral stage, non-contaminated *disposable* sterile bottles must be preferably used as the collected volumes may be lower: no CS decontamination is necessary (38). Then, during the 1st days when additional stimulation is performed with the breast pump, mothers are usually in maternity and use disposable materials already sterile. The question may arise for the milk collected by the mothers intended to be donated to human milk banks. In general, donors are mothers who have a good milk supply and who donate their surplus milk and for whom a minimum of 100–150 ml of milk per day is requested, so it seems rare that the donors fill bottles with low volumes of milk. We evaluated on a day of pasteurization the number of bottles containing <20 ml of milk at the time of milk pooling. On 26th June 2018, from 299 milk bottles drawn and for a total volume of 38.4 L, 17 pasteurized batches were constituted. Only one bottle contained <20 ml of milk, so it seems to be an exceedingly rare case. Moreover, there is an added dilution effect during milk pooling because each batch is made from 1 to 4 L, and the dilution factor of a 20-ml bottle is 50–200. Finally, extreme reactivity of HCA makes highly improbable any persistence of free HCA after addition of milk. For all these reasons, exposure to free HCA is extremely low.

THM are widespread and mildly lipophilic compounds that result from water chlorination when organic matters are present. Chronic ingestion or inhalation of THM may be dangerous because of possible toxicity and carcinogenicity of these compounds (29, 39). Drinking water can contain from 10 to 100 μg/l of THM (25). Indeed, the hydrophobic properties of THM facilitate their accumulation in milk fat. Pellizzari et al. (40) detected chloroform and dibromochloromethane in mother's milk.

More recently, Batterman et al. found 15% more THM in women's milk than cow's milk. The second part of our study showed that the THM levels in bottles decontaminated with Milton® is well below the toxic threshold (41). Atkinson and Begg (36) demonstrated that lipid content was a determining factor in the variability of THM content in both human and cow's milk (36). We did not consider this factor, but the rapid clearance of THM makes such an assessment difficult and reduces the exposure anyway (20). It is also a protective element against the risk of contamination of small volumes of breast milk production in the first few days after birth because of the low lipid content of colostrum and early lactation milk (38). This remains true for all lipophilic contaminants (42). Chlorine solution is the oldest method recognized to be efficient against bacteria including sporulate bacteria, and more recently, efficiency was confirmed by Chen et al. (2) against SARS-CoV-2. The aim of our study was not to confirm once again this efficiency of the CS but to answer to another question: how safe would it be to use it in the conditions associated with the decontamination of breast pump milk. Our data clearly demonstrate safety, which could have a major impact for a better and more extended use of these pumps.

Cold decontamination based on CS was not the method of reference before the COVID-19 pandemic (1, 33, 43). The alternative process proposed is the heat decontamination. Breast shields and bottles must boil for 20 min. However, boiling includes several limitations and risks. It is considered long with a risk of burns during handling. The material degrades very quickly with the heat, the thread is no longer waterproof, and the plastic becomes sticky. Chemical molecules can migrate from plastic to milk. Potentially released molecules include endocrine disruptors such as bisphenols or phthalates (6, 40, 42, 44, 45). In addition, there is a risk of recontamination of the equipment if it is not used directly and is stored in a non-perfectly clean or humid environment (dishcloth, dish drainer, or edge of the sink) (6, 33). Other decontamination techniques may also be considered, such as microwaves if available (20, 23–25), to increase the compliance of mothers with recommendations (4, 46), but their efficacy as a barrier measure against SARS-CoV-2 are less established.

On the other hand, CS were used by 61% of English hospital units in 2003 (35% in 2014) as a breast pump milk collection kit decontamination technique (4). Contrary to other methods, decontamination using CS avoids heat burnings and represents the advantage of being less restrictive since the material is directly usable at the time of milk collection after being submitted to a 15-min or more soaking solution. It is also the only alternative for mothers in precarious situations without electricity in their social housing (1, 32, 47).

Our study showed that when the decontamination of the breast pump milk collection kit is necessary, for premature newborns and/or with pathology, CS can be used without toxicological risk and reduce the risk of bacterial and viral contaminations of milk (26, 48, 49). With the actual COVID-19 crisis, it is interesting to point out the fact that CS is an effective method of cleaning to prevent environmental contamination (1, 43, 50, 51).

No studies had ever evaluated the toxicological safety of cold decontamination with chlorinated solutions. In view of our results and recommendations for the use of CS already published (5, 16), we recommend the following:

- Breast pump milk collection kit must be completely dismantled and cleaned with detergent before being decontaminated (1, 47).
- The manufacturer recommended dilution by must be strictly respected.
- The CS must be prepared in plastic or glass container only. Metallic container must be avoided.
- The container must be cleaned on a daily basis to prevent limescale deposits.
- Pre-cleaned items must be thoroughly immersed in the CS for the period recommended by the manufacturer and can be preferably left soaking between two uses.
- The decontaminated material should neither be rinsed with sterile or boiling water nor wiped but must be well-drained. Rinsing and wiping are no longer necessary because the chlorine residues remaining after decontamination with a properly diluted CS are negligible as we have just demonstrated. In addition, rinsing and wiping are at high risk of re-contamination.

Rejection for bacteriologic contamination is the most important risk for human milk bank. It is the reason why decontamination of breast pump is recommended at home and that single-use dispositive is used in hospital. With the COVID-19 pandemic and knowing that SARS-CoV-2 could be transmitted by hand–air contact, health professional must edit the existing guidelines to prevent milk contamination, particularly when breast milk is breast pumped for extremely vulnerable babies and chlorine solution appears to be both safe and efficient. Lastly, we propose a summary table of hygiene rules that should be respected in collection of breast milk intended to be administered, raw or pasteurized, to vulnerable newborns (Table 2) (35, 43–45). Clear and illustrated instructions on both cleaning and decontaminating breast pump milk collection kits must be provided for mothers. A demonstration session with a trained member of staff should be held (47). Breastfeeding is possible for COVID-19(+) mothers but requires barrier measures to care for the baby, breastfeed or milk draw (1, 2). On the other hand, in case of suspicion or of a confirmed case of infection with COVID, mothers will have to take strict measures to prevent airborne contamination by wearing a mask properly and washing hands. Viral RNA content (PCR) is very high between day 1 and 5 in the nasopharynx and oropharynx and becomes negative after day 14, knowing that the live virus is isolated in 83% of samples for 7 days but no longer after day 8 despite high viral loads (49). To get rid of any contamination from mother to child, a rigorous hand hygiene with liquid soap is required before and after the care of her baby or expressing milk (26). The usual recommendations are a wash of at least 30 s and drying with a disposable wipe, but the most recent data shows that the virus becomes undetectable after 15 min; hydroalcoholic solution appears to be a more efficient and rapid solution but again contact viral load modifies the data (31). If breastfeeding a newborn during the COVID-19 pandemic is not questionable, mothers must follow specific measures and barriers for herself and her environment when she breast pumps milk. The milk pump and nipples should be cleaned with a virucidal detergent/disinfectant. Moriarty et al. found virus on uncleaned surfaces 17 days after the presence of infected subjects (51). Chin et al. (30) gives us an anthology of the durations at the end of which the SARS-Cov-2 is no longer detectable: 3 h on paper and paper towels, 48 h on wood and clothing, 4 days on glass, and 7 days on plastic. Regular cleaning and disinfection of affected surfaces with a standard household disinfectant containing a 1% diluted chlorine solution appears to be an essential barrier, and CS meets these efficiency criteria (1, 19). This is a good way to secure the milk collected for these vulnerable children and to allow neonatal services and milk bank to use the mother's own milk in case of infection with COVID even if mothers remain confined to her home. When mothers use, wash, and dry the breast pump eight times per day, the outside of the bottle and the entire breast pump have the same exposition to environmental risk, which is why for vulnerable babies sterile device bottles and breast pumps are used and also why these devices should be decontaminated when sterile ones are not available. It would be a paradox to authorize devices that were not decontaminated for milk collected at home whereas only sterile devices can be used in the hospital settings.

**Table 2:** Synopsis: hygiene rules of breast milk collection for vulnerable newborn.

	**Home**	**Maternity unit**	**Neonatal unit**
Daily shower	X	X	X
Hand hygiene (liquid soap, all-purpose wipes)	X	X	X
Breast hygiene (liquid soap, all-purpose wipes)	X	X	X
Washed Breast pump kit	X		
Decontaminated breast pump kit (boiling water 20 min, chlorine solutions)	X		
Single-use breast pump kit		X	X
Sterile bottles	X	X	X
Storage at +4°C for 48 h	X	X	X
Storage at −18°C for 4 months	X for consumption after leaving the hospital		X if the milk is then pasteurized by the human milk bank

*Breast pump kit contains breast shield, collection bottle, and tubing*.

*The tubing should not be decontaminated as it contains an anti-reflux valve*.

Her milk will be even more valuable since it will be the only link between her and her child and be able to guarantee to the teams of NICU that an effective and secure method for the collection of milk is an unmissable step.

## Conclusion

This study provides reassuring data about the safety of cold decontamination with CS of breast pump milk collection kit, as residual HCA or THM concentrations were well below the guidelines. Its effectiveness has been demonstrated by other studies (1, 32). The interest to authorize and privilege its use is to limit the risk of contamination of raw milk intended to feed the most vulnerable newborns, when it is known that each stage of handling and transport can increase the risk and that the bacteriology fluctuates in such a way that the bacteriological analyses can be falsely reassuring. Thus, cold decontamination reduces losses of milk for bacteriological reasons in human milk banks (25, 33). Where disinfection of the kits and associated items is necessary, the working group recommends that heat disinfection is the method of choice, and in hospital, chemical disinfection should only be used if the local IPCT has approved the method and quality assurance of its use (32). The topic of decontamination of breast pumps and milk storage containers is relevant, since, if misinterpreted, it must be clearly explained so that it could not reduce the adherence of mothers of preterms to breast pump their own milk and of other women who donate milk to the human milk banks. Our results therefore recommend the following:

- Promote breast feeding and breast pumping even if sanitary crisis imposes hygienic measure
- Propose between different methods of chlorine solution decontamination and demonstrate its safety.

The manuscript clearly states the fact that rejection for bacteriologic contamination is the most important risk for human milk bank. It is the reason why decontamination of breast pump is recommended at home and that single-use dispositive is used in hospital. With the COVID-19 pandemic, it is crucial to reinforce that it is still not clear whether SARS-CoV-2 can or cannot be transmitted through breast milk. Even if RNA was detected in breast milk, it is not a proof of viable and infective virus whereas air and hand transmission is certain. Consequently, health professionals must edit the existing guidelines to prevent milk contamination, particularly when breast milk is breast pumped for extremely vulnerable babies. The aim of our study is not to reduce the adherence to breastfeeding but to reassure health professionals and governmental instances on the possibilities to pursue breast pump without risk. Our results could also be interesting for women living in precarious conditions or women who breast pump their milk at work who do not have any other choice for decontamination, especially the ones that do not have access to boiling water.

CS could also be of interest to prevent milk contamination of equipment used to breast pump milk by viruses like SARS-CoV-2 during crisis to prevent a shortage of human milk and to guarantee safety in the collection of the mother's own milk (1). Buosenso et al. (50) have recently reported the fact that newborns of mothers with COVID-19 can acquire the infection later after birth and pointed that the long-term consequences are still unknown, so all efficient and secure preventive measures of contamination like CS for breast pump accessory are important (52).

Thus, we do need an action to protect the environment and also target the protection of precarious newborns. We recommend using CS under the dilution conditions recommended by the manufacturer, draining normally with agitation, and not rinsing or wiping to prevent re-contamination (53). An interest is also to reduce the use of single-use equipment, a major source of plastic waste, and to offer an alternative to the use of heat when it is not possible.

## Data Availability Statement

The original contributions presented in the study are included in the article/supplementary material, further inquiries can be directed to the corresponding author/s.

## Author Contributions

All authors listed have made a substantial, direct and intellectual contribution to the work, and approved it for publication.

## Conflict of Interest

The authors declare that the research was conducted in the absence of any commercial or financial relationships that could be construed as a potential conflict of interest.

## Abbreviations

CS: chlorine solution
HCA: hypochlorous acid
THM: total trihalomethanes
CNSF: Collège National Sage Femme
CNGO: Collège National Gynécoobstétricien
WHO: World Health Organization
EMBA: European Milk Bank Association
DHM: donor human milk
NICU: neonatal intensive care unit
TDI: theorical infant dose.
